# Hospital Admissions of Huntington's Disease Patients in a Huntington's Disease Centre Between 2011 and 2016: A Retrospective Analysis

**DOI:** 10.1002/mdc3.13459

**Published:** 2022-05-05

**Authors:** Marina Peball, Beatrice Heim, Philipp Ellmerer, Florian Frank, Nadia Busin, Matyas Galffy, Atbin Djamshidian, Klaus Seppi

**Affiliations:** ^1^ Department of Neurology Medical University of Innsbruck Innsbruck Austria; ^2^ University Hospital of Psychiatry II, Department of Psychiatry, Psychotherapy and Psychosomatics Medical University of Innsbruck Innsbruck Austria

**Keywords:** Huntington's disease, hospital admissions, optimal symptom control

## Abstract

**Background:**

Huntington's Disease (HD) is a relentlessly progressive genetic neurodegenerative disorder with characteristic motor, psychiatric, and behavioral abnormalities that inevitably results in severe disability and death. Many patients have multiple hospital admissions during the disease course, but there is limited information which problems lead to hospitalization.

**Objectives:**

To assess acute reasons for hospital admissions, discharge routes, and clinical characteristics of HD patients in a retrospective analysis.

**Methods:**

We reviewed all medical records of patients with an established diagnosis of Huntington's Disease and hospital admissions between 2011 and 2016 in our local hospital‐based database.

**Results:**

There were 135 hospital admissions in 53 HD patients during the review period, representing a median of two admissions per patient. Median duration of hospitalization was seven days. The most frequent reason for admission was a worsening of HD motor symptoms (n = 77, 57.0%) such as chorea, parkinsonism, gait problems, falls, and dysphagia. Psychiatric symptoms related to HD were the second most common reason for admission (n = 58, 43.0%). Infections (including aspiration pneumonia) and traumas/surgical procedures were only responsible for 6.7% and 5.9% of admissions, respectively. Emergency admissions were not common (42.2%), and the majority of patients were able to return to their previous residency upon discharge (85.2%, home or nursing home). Recurrent admissions were associated with worse motor function and functional capacity.

**Conclusions:**

Worsening of motor and psychiatric symptoms associated with Huntington's Disease were the most common reasons for hospital admissions. Therefore, our data highlight the importance of optimal symptom control in HD patients.

Huntington's Disease (HD) is an incurable neurodegenerative disorder caused by autosomal dominantly inherited elongation of cytosine‐adenine‐guanine (CAG) repeats in the Huntington gene on chromosome 4p16.3. Patients present with motor, behavioral, and psychiatric abnormalities, whereas the latter may appear many years before age of onset as defined by the first occurrence of typical motor abnormalities.^1^, ^2^ Progressive disability in HD patients is driven by the development of severe motor symptoms such as disabling chorea, bradykinesia, rigidity, dystonia, and dysphagia, as well as cognitive deficits, functional incapacity, and dependency in activities of daily living (ADLs).^1^, ^3^ Falls, weight loss, and aspiration pneumonia are well known consequences that may require attention of a specialist in an emergency setting.^3^ However, only very few studies have systematically assessed hospital utilization among the HD population.^4^, ^5^, ^6^

In this retrospective analysis, we aimed to identify acute reasons for hospital admissions of adult HD patients treated at the Medical University of Innsbruck and its associated clinics as well as their baseline and clinical characteristics.

## Methods

We performed a retrospective analysis of all symptomatic patients with a genetically confirmed diagnosis of HD that were hospitalized at the Medical University of Innsbruck and its associated clinics between the years 2011 and 2016. All these clinics are located in the center of Tyrol and belong to the Medical University of Innsbruck, the Psychiatric Hospital in Hall, and the District Hospital in Hall. The Medical University of Innsbruck is the largest clinic in our catchment area in western Austria and the neurological Department has a specialized HD center. Patients are also referred from eastern Austria, Bavaria, and South Tyrol. The Austrian health care system is predominantly financed publicly with inpatient stays mostly receiving funding from the provinces, national government, or local authorities. Other financial compensation comes from social insurance institutions (tax revenues), patients themselves, or private health insurances. The payment calculations are based on procedure‐oriented diagnosis‐related case groups (in German: “Leistungsorientierte Diagnosen‐Fallgruppen,” LDF), i.e., an Austrian Diagnosis‐related Group (DRG) system that considers medical procedures carried out, the respective International Classification of Diseases (ICD)‐10 diagnosis, age of patients, and the hospital departments involved. Reimbursement for the duration of inpatient stay is capped.

For this retrospective study, data from our hospital database and the observational REGISTRY study of the European Huntington's Disease Network (EHDN) were used to assess baseline and clinical characteristics of HD patients at admission.^7^ For further details on the REGISTRY study, please refer to the Supplementary material. The REGISTRY study and this retrospective data analysis were approved by the local ethics committee. Data collection, interviews, and examinations were carried out in accordance with the principles expressed in the Declaration of Helsinki.

### Procedures

MP, NB, and MG extracted data from the medical records of all HD patients hospitalized in the respective time frame. Primary categories of interest were admission (emergency admission, high‐priority admission) and discharge date and route, medical discipline, the primary symptoms on admission, the main procedures conducted, comorbidities and concomitant medication, and the final discharge diagnoses according to ICD Clinical Modification codes. Primary reasons for admission were classified into five categories: “Infection (including aspiration pneumonia),” “worsening of neurological symptoms,” “worsening of psychiatric symptoms,” “surgery/traumatology,” and “other reasons.” For characterization of the patient's motor symptom burden and functionality in ADLs at the time of admission, parts of the Unified Huntington's Disease Rating Scale (UHDRS)^8^ were obtained from the REGISTRY study visit closest to the hospitalization (60.0%) or a respective UHDRS assessment (i.e., during an outpatient visit/ at admission, 40.0%). Disease stages of HD patients were determined as mild, moderate, or severe with respect to function in ADLs and the Total Functional Capacity (TFC) score.^4^, ^9^, ^10^ Comorbidities were evaluated using the Charlson Comorbidity Index (CCI), an index developed to measure overall chronic disease burden longitudinally and to predict survival.^11^

### Statistical Analyses

Categorical variables were given in number (n) and percent of the category. For quantitative measures the mean with its standard deviation and the median were calculated. Where appropriate, the interquartile range (IQR, P25–P75) was given. Integer data (length of stay, number of medications, CAG repeats) is presented as median (interquartile range and range). Descriptive analyses were performed on baseline, demographic, hospital admission, and discharge data as well as the UHDRS Total Motor Score (TMS), TMS—Chorea subscore, TFC, Functional Assessment (FA), and Independence Score (IS). The Chorea subscore was calculated as the sum of all items on chorea of the UHDRS TMS (face, bucco‐oro‐lingual region, trunk, both upper and lower extremities). Parametric and nonparametric tests were used for statistical analyses depending on the distribution and the scale type of the variables (see table legends). Spearman's rank‐order correlation (*r*_s_) was used to test for any relationship between discharge route (home, nursing home placement, new nursing home placement) and total number of hospital admissions per person and any relationship between the latter and total number of visits to the neurological outpatient department in the time‐period. The significance level was set at p ≤ 0.05 for all analyses. SPSS 25.0 for windows (SPSS Inc., IBM Corporation and other(s) 1989, 2017, Chicago, IL) was used to analyze data.

## Results

In total, 161 patients with HD were treated in the specialized outpatient department between 2011 and 2016. This retrospective analysis included 53 patients (n = 36 female [67.9%], n = 52 Caucasian [98.1%]) with 135 hospital admissions over six years. Respectively, 32.9% of all HD patients seen in the outpatient department required a hospital admission. Per year, almost a quarter of outpatients were admitted to the hospital (median 24.4%, Table S1). Interestingly, there was a weak but non‐significant association between total number of hospital admissions per person and total number of visits to the neurological outpatient department (*r*_s_ = 0.246, *P* = 0.08).

Table 1 shows demographic characteristics and clinical data of the 53 analyzed HD patients. The mean age of HD onset was 41.55 ± 10.92 (40.50) years in all patients. More than half of all patients (n = 28, 52.8%) had an advanced disease stage. The mean number of hospitalizations per patient was 2.55 ± 2.07 (2.00). Median duration of hospitalization was 7 days. Most HD patients had no HD‐unrelated comorbid diseases at the time of hospitalization with a mean CCI of 0.56 ± 1.24 (0.00). Dementia was present in 20 patients (37.7%, with 53 hospital admissions, Table 1). All of them had an advanced disease stage. Patients with dementia had significantly more emergency admissions (*P* < 0.01). No significant association was found between dementia and different admission reasons. The median number of overall medications at admission was 5.00 and 6.00 at discharge with a statistically significant difference (*P* < 0.01).

**TABLE 1:** *Demographic and clinical data of patients with Huntington*'*s disease*

Demographic data	
Sex	
Female	36 (67.9%)
Male	17 (32.1%)
Ethnicity	
Caucasian	52 (98.1%)
Other	1 (1.9%)
Age of onset (in years)	41.55 ± 10.92 (40.50)
Female	40.73 ± 9.99 (38.92)*
Male	44.27 ± 13.93 (50.92)*
CAG repeat	
Short allele	18.00, P25–P75: 16.75–21.25, range: 11–38^a^
Longer allele	45.00, P25–P75: 43–47, range: 40–72
First symptoms	
Motor	29 (60.4%)
Psychiatric	11 (22.9%)
Cognitive decline	2 (4.2%)
Mixed	6 (12.5%)
Handedness	
Right‐handed	37 (90.2%)
Left‐handed	2 (4.9%)
Both sides	2 (4.9%)
Marital status	
Married/in a relationship	29 (54.7%)
Single/divorced/widowed	16 (30.2%)
Unknown	8 (15.1%)
Working status	
Working (full‐time / part‐time)	8 (3 / 5) (15.1%)
Unemployed (e.g., housewife)	8 (15.1%)
Unemployed other reasons	2 (3.8%)
Retired due to age	7 (13.2%)
Retired due to HD	27 (50.9%)
Sick leave	1 (1.9%)
Family history	
Mother affected	19 (35.9%)
Father affected	22 (41.5%)
No affected parent or unknown	12 (22.6%)
Mean age of onset in affected parent (in years)	
Affected mother	46.00 ± 12.46 (40.00)
Affected father	46.00 ± 10.66 (40.00)
Disease stages	
Mild	13 (24.5%), Female: 9 (25.0%)**
Moderate	12 (22.6%), Female: 7 (19.4%)**
Severe	28 (52.8%), Female: 20 (55.6%)**
Number of hospital admissions per person	2.55 ± 2.07 (2.00)
Female	2.44 ± 1.76 (2.00)
Male	2.76 ± 2.66 (2.00)

Clinical data	
Age at admission (in years)	53.00 ± 14.57 (50.08)
Disease duration until admission (in years)	11.69 ± 5.84 (11.75)
Length of stay (in days)	7.00, P25–P75: 4–13, range: 1–348
BMI at admission (in kg/cm^2^)	22.43 ± 3.79 (22.39)
Comorbidities at admission (CCI)	0.56 ± 1.24 (0.00) (P25–P75: 0.00–1.00)
Number of medications at admission	5.00, P25–P75: 3–7, range: 0–15*
Number of medications at discharge	6.00, P25–P75: 4–8, range: 0–16*
UHDRS TMS at admission	50.32 ± 19.39 (53.00)
UHDRS TMS—Chorea subscore at admission	9.13 ± 6.75 (7.00)
TFC at admission	4.97 ± 4.03 (4.00)
FA at admission	14.77 ± 9.12 (15.00)
IS at admission	63.73 ± 19.89 (65.00)
Dementia	20 (37.7%) patients with 53 (39.3%) hospital admissions
Recurrent falls (>1 per year)	14 patients (26.4%) with 25 (18.5%) hospital admissions

Continuous data are presented as mean ± standard deviation (median). Integer data is presented as median (interquartile range and range). For categorical data the number and percent (%) are given. If the standard deviation provides a value below zero, the interquartile range is given.

Higher scores indicate better status of the patient in the TFC, the FA, and the IS. UHDRS TMS range: 0–124 points, Chorea subscore: 0–28 points, TFC: 0–13 points, FA: 0–25 points, IS: 5%–100%.

* An unpaired *t*‐test for normally distributed continuous variables was used to assess the difference between age of onset of females and males: *P*‐value = 0.41. A paired‐samples *t*‐test was used to analyze the difference between the number of medications at admission and discharge: *P*‐value <0.01.

** No significant difference of sex distribution using Qui‐square test and Fisher's exact test.

^a^ Two patients in our database were homozygote with a CAG repeat length of 38 and 36 on the shorter allele.

Abbreviations: n, number; CAG, cytosine adenine guanine; P, percentile; kg, kilograms; m, meters; cm, centimeters; CCI, Charlson Comorbidity Index; HD, Huntington's disease; UHDRS, Unified Huntington's Disease Rating Scale; TMS, Total Motor Score; TFC, Total Functional Capacity; FA, Functional Assessment; IS, Independence Score.

Table 2 summarizes data of the 135 admissions and discharges. It must be noted that some patients had more than one primary reason for admission.

**TABLE 2:** Admission and Discharge characteristics

Admission	
Reasons for admission* (Total n = 135)	
Worsening of neurologic symptoms^a^	77 (57.0%)
Worsening of psychiatric symptoms^b^	58 (43.0%)
Other^c^	13 (9.6%)
Infection (incl. Aspiration pneumonia)^d^	9 (6.7%)
Surgery, traumatology^e^	8 (5.9%)
Admission source	
Emergency admission	57 (42.2%)
High‐priority admission	76 (56.3%)
Unknown	2 (1.5%) (to psychiatry)

Discharge	
Discharge diagnosis according to diagnostic code from hospital information system (Total n = 135)	
HD	91 (67.4%)
Psychiatric diagnoses	18 (13.3%)
Other^f^	16 (11.9%)
Dementia (in HD or not specified)	5 (3.7%)
Infection (incl. Aspiration pneumonia)	5 (3.7%)
Discharge route/death during inpatient stay	
Home	92 (68.2%)
Nursing home/long‐term care facility	27 (20.0%)
New nursing home admission	4
Other hospital	8 (5.9%)
Unknown	6 (4.4%)
Death during hospitalization	2 (1.5%)

For categorical data the number and percent (%) are given.

* Some patients had more than one reason for admission.

^a^ Frequent complaints were a worsening of chorea (n = 26, 33.8%), parkinsonism (n = 35, 45.5%), a worsening of both chorea and parkinsonism (n = 5, 6.5%), gait problems with recurrent falls, or dysphagia (n = 34/77, 44.2%).

^b^ Worsening of psychiatric symptoms included depression (46.6%), aggressive behavior (36.2%), agitation and irritability (41.4%), psychotic behavior with delusions (24.1%), or suicidal tendencies.

^c^ “Other reasons” for admission (9.6%) included dental (desolate condition of the teeth and adaption of dental prosthesis, n = 2), and dermatologic (atopic dermatitis, n = 4 in 1 patient) treatment, admission due to retinal detachment (ophthalmologist, n = 3 in 1 patient), rehabilitation after a femoral neck fracture (n = 1), atrioventricular block III (internal medicine, n = 1), social reasons (n = 1), and withdrawal of analgesic medication in an HD patient with chronic mixed headache (n = 1) (Supplementary material).

^d^ A total of 6.7% of hospital admissions (n = 9) were due to infections: one phlegmon of the floor of the mouth (ear‐nose‐throat specialist, n = 1), aspiration pneumonia (n = 5), acute sigma diverticulitis and salpingitis (n = 1), and urinary tract infection (n = 2). Aspiration pneumonia was the reason for five hospital admissions (3.7%) and one death during inpatient stay.

^e^ Surgery and traumatology (n = 8, 5.9%) admissions comprised gastrointestinal obstructive symptoms (n = 1), revision after accidental removal of the percutaneous endoscopic gastrostomy (PEG) tube (n = 1), an operation of a cerebellar angle bridge tumor (n = 1), acute fractures of the humerus and femur (n = 1 each), a polytrauma (biking accident, n = 1), a head trauma with cerebral contusions and a nasal fracture (n = 1), as well as treatment of a chronic subdural hematoma (with an acute nasal fracture, n = 1). The latter five admissions were due to injurious falls.

^f^ Other primary discharge diagnoses (11.9%) included the diagnosis of a phlegmon of the base of the mouth (n = 1), a retinal detachment (n = 3), pyoderma and folliculitis (n = 1), atopic dermatitis (n = 3), traumatic subarachnoid hemorrhage (n = 2), a perforated diverticulitis of the colon sigmoideum (n = 1), a fracture of the femur (n = 1), multiple intracranial and cranial fractures after a trauma (n = 1), a fracture of the shoulder and shoulder girdle (n = 1), a newly diagnosed atrioventricular block (n = 1), and a neurosurgical intervention for a tumor of the cerebello‐pontine angle (n = 1).

The most common reasons for admission were worsened neurological symptoms of HD (n = 77, 57.0%). Frequent complaints were a worsening of chorea, parkinsonism, gait problems with recurrent falls, or dysphagia (n = 34/77, 44.2%). Worsening of chorea was a major problem in 26 of 77 HD patients (33.8%) and worsening of akinetic‐rigid symptoms in 35 of 77 patients (45.5%). A combination of choreatic and akinetic‐rigid symptom deterioration was the reason for five of 77 neurologic admissions (6.5%). Recurrent falls (>1 in the previous year) were a major complaint in 25 hospital admissions (18.5%) in 14 patients (26.4%) including five injurious falls.

The other major reason for hospitalization of HD patients was worsening of psychiatric symptoms (n = 58, 43.0%) with depression (46.6%), aggressive behavior (36.2%), agitation and irritability (41.4%), psychotic behavior with delusions (24.1%), or suicidal tendencies (Table 2). Five hospital admissions (3.7%, in four patients) were due to acute suicidal thoughts, none because of suicide attempts. In one patient this was due to an adverse reaction to tetrabenazine. In three demented patients, delirium led to admission to a psychiatric ward. According to the medical records, the psychiatric and behavioral problems improved upon discharge. Antipsychotics were documented as pre‐existing medication in 73 (54.1%) of all admissions, antidepressants in 70 (51.9%), benzodiazepines in 62 (45.9%), amantadine in 43 (31.9%), tiapride in 5 (3.7%), tetrabenazine in 17 (12.6%), and levodopa in 10 (7.4%).

In 30 (51.7%) admissions to psychiatric wards, antipsychotics were the pre‐existing medication, antidepressants in 24 (41.4%), benzodiazepines in 20 (34.5%), amantadine in 17 (29.3%), tiapride in 2 (3.5%), tetrabenazine in 8 (13.8%), and levodopa in 2 (3.5%) (Table S2).

Intriguingly, a worsening of both neurological and psychiatric symptoms was noted in 27 (20.0%) of the hospitalizations. Table 2 also summarizes further reasons for admission including admissions for infectious diseases, surgical/ traumatological, and other reasons (table legend). Aspiration pneumonia was the reason for five hospital admissions (3.7%) and one death during inpatient stay (Table 2).

The most common relevant comorbidities in the patients hospitalized were urinary tract infections (n = 53, 39.3%) and gastrointestinal problems (i.e., esophagitis with ulcer in a patient with nausea and vomiting) (n = 5, 3.7%).

All patients received physical, occupational, and speech therapy. The most frequent consultations during inpatient stays included neuropsychologists for cognitive assessment (11.1%), physicians from the Department of Hearing, Speech and Voice Disorders (9.6%), neurologists (5.9%) (i.e., if patients were not hospitalized at a neurological ward [51.1% of all admissions]), and psychiatrists (5.2%, Table S3). A detailed list of the main procedures conducted can be found in the Supplementary material and table.

In 67.4% of all hospitalizations, the primary discharge diagnosis was HD. A primary psychiatric discharge diagnosis was given in 13.3% and 3.7% either had a diagnosis of dementia or an infectious disease (e.g., aspiration pneumonia, Table 2). Two patients died during inpatient stay, one after a polytrauma from a biking accident and another due to aspiration pneumonia. Most hospitalizations (68.2%) resulted in a discharge back home or to a nursing home (20.0%). Four patients previously living with their families were admitted to a nursing home due to overall symptom worsening (Table 2; Mean age at admission: 57.98 years ± 13.78; median length of stay: 15 days). Two of these patients had been acutely admitted (psychosis, dementia/social indication) and a worsening of neurological symptoms (motor, cognitive) was the primary reason in three of these cases. There was a significant correlation between discharge route (home, nursing home placement, new nursing home placement) and number of total hospital admissions per person (*r*_s_ = 0.293, *P* < 0.01). The four patients that were newly admitted to the nursing home had the highest number of hospital admissions respectively (6, 7, 11). In 5.9% of all admissions, a transfer to another hospital for further rehabilitation or therapy was necessary (Table 2).

HD patients who were admitted due to infections (including aspiration pneumonia) (64.44 years ± 9.55 [69.08]), or for surgeries/due to a trauma (68.26 years ± 15.66 [75.71]) were older than HD patients who were admitted due to a worsening of neurological (51.85 years ± 15.98 [52.42]) or psychiatric symptoms (50.93 years ± 12.18 [48.92]) or “other” reasons (47.72 years ± 17.38 [43.50], Table 3). Overall, surgical patients showed longer durations of inpatient stays (median: 17.50 days) than the other patient groups (neurological symptoms: 7.00 days; psychiatric symptoms: 7.00 days; “other”: 5.00 days; infections: 6.00 days). Emergency in‐patient admissions mostly occurred in patients with a worsening of psychiatric symptoms (n = 31, 55.4%), infections (including aspiration pneumonia) (n = 8, 88.9%), and a surgery/trauma (n = 6, 75.0%). All 17 HD patients admitted due to infections (including aspiration pneumonia) or to a surgical ward/traumatology had a severe disease stage (Table 3).

**TABLE 3:** *Data of Huntington*'*s disease patients with different clinical presentation at admission*

Data at admission	Neurologic symptoms (n = 77)	Psychiatric symptoms (n = 58)	Other (n = 13)	Infection (including aspiration pneumonia) (n = 9)	Surgery, Traumatology (n = 8)
Age (in years)	52.60 ± 14.83 (53.25)	50.93 ± 12.18 (48.92)	47.72 ± 17.38 (43.50)	64.44 ± 9.55 (69.08)	68.26 ± 15.66 (75.71)
Length of stay (in days)	7.00, P25–P75: 4–10, range: 1–42	7.00, P25–P75: 5–14.75, range: 1–348	5.00, P25–P75: 3–9.50, range: 1–71	6.00, P25–P75: 3.50–12, range: 3–49	17.50, P25–P75: 2.25–36.75, range: 1–52
Disease duration (in years)	11.65 ± 5.76 (11.71)	11.14 ± 5.05 (11.17)	9.51 ± 7.55 (8.58)	16.42 ± 5.71 (14.88)	16.78 ± 2.76 (17.75)
Females	59 (76.6%)	32 (55.2%)	8 (61.5%)	5 (55.6%)	6 (75.0%)
Admission					
Emergency	15 (19.5%)	31 (55.4%)	4 (30.8%)	8 (88.9%)	6 (75.0%)
High‐priority	62 (80.5%)	25 (44.6%)	9 (69.2%)	1 (11.1%)	2 (25.0%)
Disease stage					
Mild	11 (14.3%)	5 (8.6%)	4 (30.8%)	0	0
Moderate	23 (29.9%)	18 (31.0%)	0	0	0
Severe	43 (55.8%)	35 (60.3%)	9 (69.2%)	9 (100%)	8 (100%)

Continuous data are presented as mean ± standard deviation (median). Integer data is presented as median (interquartile range and range). For categorical data the number is given. If the standard deviation provides a value below zero, the interquartile range is given.

Abbreviation: P, percentile.

Patients with more than two hospital admissions (i.e., recurrent admissions) had significantly higher score values of the TFC (*P* = 0.01), FA (*P* = 0.01), IS (*P* = 0.02), and the TMS (*P* = 0.01) at their first admission compared to patients who were only admitted once or twice between 2011 and 2016 (Table 4). Moreover, patients with recurrent admissions had significantly higher disease stages (mild: 5.6%, moderate: 22.2%, severe: 72.2%) when compared to patients with ≤2 admissions (mild: 34.2%, moderate: 22.9%, severe: 42.9%; *P* = 0.05, Table 4). There were no differences in sex, age, comorbidities (CCI), or disease duration in HD patients with ≤2 and >2 hospital admissions (Table 4).

**TABLE 4:** *Clinical Characteristics of Huntington*'*s disease patients with ≤2/>2 admissions to the hospital*

	≤2 admissions (n = 35)	>2 admissions (n = 18)	*P*‐value^a^
Sex			0.89
Male	11 (31.4%)	6 (33.3%)	
Female	24 (68.6%)	12 (66.7%)	
Age at first admission (years)	52.09 ± 13.63 (51.25)	49.69 ± 20.28 (48.66)	0.61
Disease duration at first admission (years)	9.38 ± 5.71 (9.63)	11.76 ± 5.57 (11.75)	0.17
Charlson comorbidity index	0.46 ± 1.09 (0.00) (P25–P75: 0.00–1.00)	0.33 ± 0.69 (0.00) (P25–P75: 0.00–0.25)	0.66
UHDRS TMS	37.48 ± 16.26 (37.00)	51.60 ± 12.75 (50.50)	0.01
UHDRS TMS – Chorea subscore	7.30 ± 5.03 (6.00)	9.10 ± 6.39 (11.50)	0.39
TFC	8.26 ± 4.00 (9.00)	4.10 ± 2.42 (3.50)	0.01
Disease stage			0.05
Mild	12 (34.2%)	1 (5.6%)	
Moderate	8 (22.9%)	4 (22.2%)	
Severe	15 (42.9%)	13 (72.2%)	
FA	20.35 ± 8.02 (21.00)	13.60 ± 4.95 (14.00)	0.01
IS	77.11 ± 16.61 (85.00)	62.50 ± 11.37 (57.50)	0.02

Categorical data are presented as n, %. Continuous data are given in mean ± standard deviation (median). If the standard deviation provides a value below zero, the interquartile range is given.

Higher scores indicate better status of the patient in the TFC, the FA, and the IS. UHDRS TMS range: 0–124 points, Chorea subscore: 0–28 points, TFC: 0–13 points, FA: 0–25 points, IS: 5%–100%.

^a^ Unpaired *t*‐test for normally distributed continuous variables (Chorea subscore, disease duration, Charlson Comorbidity Index, age at admission), Mann–Whitney U test for not normally distributed continuous variables (TMS, TFC, FA, IS). Chi‐square test for categorical data.

Abbreviations: n, number; UHDRS, Unified Huntington' Disease Rating Scale; TMS, Total Motor Score; TFC, Total Functional Capacity; FA, Functional Assessment; IS, Independence Score.

## Discussion

Here, we performed a retrospective analysis of 135 hospital admissions in 53 HD patients at the Medical University Innsbruck, Austria.

The most frequent reason for hospitalization was a worsening of motor symptoms of HD including chorea, parkinsonism, gait problems, falls, and dysphagia. Interestingly, in a total of 25 hospital admissions in 14 patients (26.4%), recurrent falls were a major complaint, with five of them being injurious falls requiring medical treatment. Indeed, recurrent falls are common in the HD population with a one‐month incidence of 28.8%.^12^ Psychiatric symptoms related to HD were the second most common reason for admission and included depression with five admissions due to suicidality, aggressive behavior, agitation and irritability, psychotic behavior with delusions, or delirium. The percentage of suicidal ideations in our cohort is consistent with previous studies.^13^ As expected for psychiatric admissions, the most common medication change was the introduction or change of antipsychotics and benzodiazepines as well as dose increase of antidepressants and dose reduction or discontinuation of amantadine.

Surprisingly, infections (including aspiration pneumonia) and traumas/ surgical procedures were only responsible for 6.7% and 5.9%, respectively. Despite dysphagia being a common reason for admission, aspiration pneumonia was a relatively uncommon reason for hospitalization (3.7%) compared to other HD studies (42–44%).^14^, ^15^, ^16^, ^17^ An explanation for this may be that advanced HD patients with pneumonia are more often referred to a local hospital. Moreover, our advanced HD patients often receive a palliative care plan for treatment at home or are placed in a long‐term care facility.

Besides physical, occupational, and speech therapy, the most frequent consultations during inpatient stays were neuropsychologists, physicians from the Department of Hearing, Speech and Voice Disorders, neurologists (i.e., if patients were not hospitalized at a neurological ward), and psychiatrists. The number of long‐term medications also changed significantly during hospitalization, both reflecting (motor) symptom burden and the need of therapeutic re‐evaluation.

During the hospital stay, many of our patients were treated for well‐known comorbidities and complications of HD such as gastrointestinal problems, urinary tract infections, dental complications, neurosurgical problems, or ophthalmological diseases.^18^, ^19^, ^20^, ^21^, ^22^, ^23^ Most patients in our study were discharged with the primary diagnosis of HD, dementia associated with HD, or psychiatric symptoms associated with HD undermining the debilitating influence of the disease on the patient's life.

By contrast to our findings, one American study found respiratory diseases (22.0%) and sepsis (11.2%), psychiatric symptoms (21.5%), and malnutrition, dehydration, and worsening of skin condition (15.5%) to be common reasons for hospital admissions in HD patients with similar age, but more advanced disease stages. Data in this study, however, were derived from the Healthcare Cost and Utilization Project (HCUP, Agency for Healthcare Research and Quality, AHRQ) which excludes federal, primary psychiatric, rehabilitation, or long‐term care hospitals.^4^ The divergent reasons for hospital admissions compared to our study might be caused by differences of data acquisition and healthcare systems (USA versus Austria) as well as inclusion of more advanced HD patients in the American study.^4^ Of note, the number of admissions due to a trauma (5.7%) was comparable to our data.^4^

Another more recent study assessed reasons for hospital admissions in juvenile HD patients (here defined as people with HD aged ≤20 years) and found epilepsy, seizures, and convulsions to be most frequent (58.5%), followed by psychiatric symptoms (26.1%), and respiratory infections (15.9%).^5^ There were only two patients (3.8%) with pediatric HD (motor onset <18 years)^24^, ^25^ in our cohort (CAG repeats on the longer allele: 70 and 72). By contrast to a pediatric onset of HD, seizures are an uncommon presentation in adult‐onset HD. Therefore, a different clinical presentation of adult HD patients compared to patients with a younger age of onset^26^, ^27^ is the most likely explanation of the discrepant results.

Most of our patients with worsening of neurologic or psychiatric symptoms could be discharged home, confirming the findings of an English study assessing HD patient's discharge routes from a psychiatric ward.^6^

Fifty‐three percent of all HD patients in our study were at an advanced disease stage, more common in the group with recurrent (>2) inpatient stays. Moreover, patients with recurrent admissions showed significantly higher dependency, functional impairment, and overall motor symptom burden (all *P* ≤ 0.02). Sex, disease duration, age at first admission, and comorbidities were not associated with recurrent admissions. Thus, motor symptoms with subsequent limitations in daily functioning were not only the most prominent reason for HD patient's hospital admission but also resulted in repeated inpatient stays. This emphasizes the need for therapeutic re‐evaluation and organization of adequate assistance at home, which is regularly taken care of during inpatient stays at our clinics. The multidisciplinary treatment approach for motor impairment based on medication adjustments and exercise/intensive neurorehabilitation led to an improvement of motor symptoms during the hospital stay as reported by the patients and caregivers upon discharge from the neurologic and psychiatric wards. Different approaches have been evaluated in other countries: regular visits by a specialized HD nurse for example reduced the number and duration of hospital admissions in New Zealand significantly and remaining hospitalizations were more appropriate according to the authors.^28^

Conclusion

This is one of only very few studies systematically assessing hospital admission in HD patients. Our data suggest that adult HD patients are predominantly admitted to the hospital due to a worsening of HD motor symptoms that result in limitations in daily functioning. The second most common reasons for admission in HD patients were psychiatric symptoms. Admissions due to traumas or infections (including aspiration pneumonia) as well as emergency admissions were less common, and discharge to the patient's previous residency was possible in most cases. Our data highlight the importance of regular and careful neurological and psychiatric evaluation of HD patients, ideally in a specialized HD center, as well as optimal motor symptom control. In HD patients with debilitating symptoms, therapeutic re‐evaluation may be necessary for treatment optimization with optimal monitoring of drug response and adverse events.

## Author Roles

1) Research project: A. Conception, B. Organization, C. Execution; 2) Statistical Analysis: A. Design, B. Execution, C. Review and Critique; 3) Manuscript: A. Writing of the first draft, B. Review and Critique.

MP: 1B, 1C, 2A, 2B, 3A

BH, AD: 1C, 2C, 3B

PE, FF: 2C, 3B

NB: 1B, 1C, 3B

MG: 1C, 2C, 3B

KS: 1A, 1B, 1C, 2A, 2B, 2C, 3B

## Disclosures

### Funding Sources and Conflicts of Interest

The European Huntington's Disease Network Registry Study was funded by the CHDI. The study center received an unrestricted grant from AOP Orphan Pharmaceuticals AG for academic research in HD patients.

### Financial Disclosures for the Previous 12 Months

MP, PE, NB, MG, and AD declare that there are no additional disclosures to report.

FF received honoraria from Novartis AG and Teva Pharmaceutical Industries Limited outside the submitted work. BH reports honoraria from Novartis AG and AOP Orphan Pharmaceuticals AG outside the submitted work. Klaus Seppi reports personal fees from Teva, UCB, Lundbeck, AOP Orphan Pharmaceuticals AG, Roche, Grünenthal, Stada, Licher Pharma, Biogen, BIAL and Abbvie, honoraria from the International Parkinson and Movement Disorders Society, research grants from FWF Austrian Science Fund, Michael J. Fox Foundation, and AOP Orphan Pharmaceuticals AG, outside the submitted work.

### Ethical Compliance Statement

The REGISTRY study and this retrospective data analysis were approved by the ethics committee of the Medical University of Innsbruck. All patients or their legal representatives gave written informed consent prior to participate in the REGISTRY study. For the retrospective data analysis, informed patient consent was not necessary. On behalf of all co‐authors, the first and corresponding author confirm that all authors have read the Journal's position on issues involved in ethical publication and affirm that this work is consistent with those guidelines.

## Supporting information


**Appendix S1** Supporting information.
