# Supplementary material for: Hospital Admissions of Huntington's Disease Patients in a Huntington's Disease Centre Between 2011 and 2016: A Retrospective Analysis
Source: Mov Disord Clin Pract. 2022 May 5;9(5):628–36. doi: 10.1002/mdc3.13459 (PMC9274354; doi:10.1002/mdc3.13459)
Supplement: Supplementary file 1 — Appendix S1 Supporting information. [file MDC3-9-628-s001.docx]

**Supplementary Material**

**Hospital Admissions of Huntington´s Disease patients in a Huntington´s Disease centre between 2011 and 2016: a retrospective analysis**

Marina Peball^1^ MD, PhD, Beatrice Heim^1^ MD, PhD, Philipp Ellmerer^1^ MD, Florian Frank^1^ MD, PhD, Nadia Busin^1^, Matyas Galffy^2^ MD, Atbin Djamshidian^1*^ MD, PhD, and Prof. Klaus Seppi^1*^ MD.

*^1^ Department of Neurology, Medical University of Innsbruck, Anichstraße 35, 6020 Innsbruck, Austria*

*^2^ University Hospital of Psychiatry II, Department of Psychiatry, Psychotherapy and Psychosomatics, Medical University of Innsbruck, Anichstraße 35, 6020 Innsbruck, Austria*

**Shared corresponding authors*

Running title: **Hospital admissions in Huntington´s Disease**

**Corresponding authors:**

Klaus Seppi, MD

Department of Neurology, Innsbruck Medical University,

Anichstraße 35, A-6020 Innsbruck, Austria,

Tel +43/512/504/24279,

Fax +43/512/504/23852,

E-mail: [Klaus.Seppi@tirol-kliniken.at](mailto:Klaus.Seppi@tirol-kliniken.at)

Atbin Djamshidian, MD, PhD

Medical University of Innsbruck, Department of Neurology

Anichstraße 35, 6020 Innsbruck, Austria

Tel.: +43/512/504/25810

Fax: +43/512/504/25819

E-mail: [Atbin.Djamshidian-tehrani@i-med.ac.at](mailto:Klaus.Seppi@tirol-kliniken.at)

# Supplementary: Methods

## Study Participants and Ethics

The REGISTRY study (ClinicalTrials.gov Identifier: NCT01590589) was an observational study initiated by the European Huntington´s Disease Network (EHDN) to collect demographic and clinical data of patients with signs and symptoms of HD, pre-symptomatic gene carriers, and individuals from HD families with an unknown carrier status or a negative genetic test result.^1^ Exclusion criteria were incapability or unwillingness to take part. The REGISTRY study was completed at our centre in 2016. All patients or their legal representatives gave written informed consent prior to participation in the REGISTRY study. The interviews and examinations were carried out in accordance to the principles expressed in the Declaration of Helsinki.

## Procedures

To identify admissions of HD patients to the Medical University of Innsbruck, the Psychiatric Hospital in Hall, and the District Hospital in Hall between the years 2011 to 2016, we initially performed an automated search of our hospital database using the diagnosis “HD” or its International Statistical Classification of Diseases and Related Health Problems (ICD) code. Moreover, we manually searched for hospitalizations in medical records of all HD patients that were seen in our specialized outpatient department in the respective time frame and the year before (MP, NB). Data were extracted manually from medical records of the identified cases and data accuracy was verified by a second reviewer (MP, NB, MG).

Baseline data from medical records and the REGISTRY study (date of birth, sex, handedness, weight and height at admission, age of onset i.e., appearance of first symptoms, first symptoms, date of diagnosis, genetic test results, family and medication history, marital and working status) were merged and data integrity validated. All but seven patients had their REGISTRY visit (60.0%) or a respective Unified Huntington´s Disease Rating Scale (UHDRS) assessment (i.e., during an outpatient visit / at admission, 40.0%) within three months prior or after their respective hospital admissions. Five of these seven patients were admitted on a Psychiatric Ward and the other two patients were admitted because of trauma. In these patients, the respective REGISTRY data were beyond three months (± 7 months).

Admission source was coded to emergency or high-priority and discharge routes comprised discharge to the patient´s home, a nursing home, or another hospital. Disease duration until admission and length of stay were calculated. The number of medications on admission and at discharge was calculated, including enteral tube feedings and high caloric drinks.

# Supplementary: Other results

About 10% of patients (n=13) were admitted due to other reasons as predefined. These included dental (n=2), and dermatologic (atopic dermatitis, n=4) treatment, admission due to retinal detachment (ophthalmologist, n=3), rehabilitation after a femoral neck fracture (n=1), atrioventricular block III (internal medicine, n=1), and withdrawal of analgesic medication in an HD patient with chronic mixed headache (n=1). One patient was admitted for social reasons (organisation of social support due to a complete dependency in ADLs (n=1).

Supplementary Table 3 presents **main procedures conducted** during inpatient stays. Thirty-six % of all hospital admissions included performance of an electrocardiogram. In 52.6% of hospital admissions imaging was performed with x-ray being the most frequent (24.4%) followed by cerebral computed tomography (11.1%). In almost two-thirds of all hospital admissions (62.2%) no consultant from another specialty was needed. Other assessments including electroencephalogram or polysomnography were rare (6.7%).

Supplementary Table 1: Number of hospitalizations compared to outpatient visits of Huntington´s disease patients at the Medical University of Innsbruck

| **Year** | **Patients seen at the outpatient department (n)** | **Patients that were hospitalized (n)** | % * |
| --- | --- | --- | --- |
| 2011 | 55 | 15 | 27.3 |
| 2012 | 60 | 17 | 28.3 |
| 2013 | 64 | 11 | 17.2 |
| 2014 | 64 | 10 | 15.6 |
| 2015 | 70 | 15 | 21.4 |
| 2016 | 78 | 24 | 30.8 |

Abbreviation: n, number. Double counting of patients was avoided.

* Proportion of hospitalized patients compared to patients seen at the outpatient department.

Supplementary Table 2: Changes in drug classes during hospitalizations for neurological and psychiatric reasons

| **Drug class** | | **at admission** | **at discharge** | **dose increase** | **dose decrease** | **Change within drug-class** | **Comment** |
| --- | --- | --- | --- | --- | --- | --- | --- |
|  | | **Admissions for neurological reasons (n=77)** | | | | | |
|  | | **admissions in patients with a worsening of chorea (n=26)** | | | | | |
| Tiapride | | 3 | 4 | 0 | 1 | NA | Dose decreased due to apathy |
| Tetrabenazine | | 7 | 10 | 2 | 2 | NA | Dose decreased due to depression |
| Antipsychotics | | 12 | 16 | 2 | 0 | 4 |  |
|  | Olanzapine | 5 | 6 | 1 | 0 | NA |  |
|  | Amisulpirid | 4 | 5 | 1 | 0 | NA |  |
|  | Risperidon | 3 | 6 | 0 | 0 | NA |  |
|  | Quetiapine | 2 | 1 | 0 | 0 | NA |  |
| Benzodiazepines | | 6 | 7 | 1 | 1 | 1 |  |
| Antidepressants | | 18 | 17 | 1 | 0 | 0 |  |
| Amantadine | | 11 | 16 | 0 | 4 | NA | Dose decreased due to agitation, irritability and aggression |
| Levodopa | | 0 | 0 | 0 | 0 | NA |  |
| Cannabinoids | | 5 | 8 | 1 | 0 | NA |  |
|  | | **admissions in patients with a worsening of parkinsonism (n=35)** | | | | | |
| Tiapride | | 0 | 0 | 0 | 0 | NA |  |
| Tetrabenazine | | 7 | 5 | 1 | 4 | NA | Dose increased in a patient where antipsychotic dose was decreased |
| Antipsychotics | | 18 | 17 | 4 | 4 | 3 |  |
|  | Olanzapine | 8 | 5 | 0 | 2 | NA |  |
|  | Amisulpirid | 5 | 6 | 2 | 0 | NA |  |
|  | Risperidon | 6 | 5 | 0 | 2 | NA |  |
|  | Quetiapine | 2 | 4 | 2 | 0 | NA |  |
| Benzodiazepines | | 21 | 19 | 4 | 2 | 3 |  |
| Antidepressants | | 24 | 24 | 3 | 1 | 0 |  |
| Amantadine | | 7 | 7 | 1 | 0 | NA |  |
| Levodopa | | 8 | 10 | 1 | 3 | 0 | In all patients with dose decreases, cannabinoids were introduced due to concomitant dystonia. |
| Cannabinoids | | 3 | 13 | 0 | 0 | NA |  |
|  | | **admissions in patients with a worsening of chorea and parkinsonism (n=5)** | | | | | |
| Tiapride | | 1 | 1 | 0 | 1 | NA | Dose decreased due to depression and gait disorder |
| Tetrabenazine | | 1 | 2 | 0 | 1 | NA | Dose decreased due to gait problems with freezing of gait |
| Antipsychotics | | 4 | 4 | 0 | 2 | 2 | Dose decreased due to gait problems with falls |
|  | Olanzapine | 2 | 1 | 0 | 1 | NA |  |
|  | Amisulpirid | 0 | 1 | 0 | 0 | NA |  |
|  | Risperidon | 2 | 1 | 0 | 1 | NA |  |
|  | Quetiapine | 0 | 1 | 0 | 0 | NA |  |
| Benzodiazepines | | 1 | 2 | 0 | 0 | 0 |  |
| Antidepressants | | 5 | 5 | 1 | 1 | 0 |  |
| Amantadine | | 2 | 2 | 0 | 0 | NA |  |
| Cannabinoids | | 0 | 2 | 0 | 0 | NA |  |
| Levodopa | | 1 | 0 | 0 | 0 | NA |  |
|  | | **Admissions for psychiatric / behavioral reasons (n=58)** | | | | | |
| Tiapride | | 2 | 4 | 0 | 1 | NA | Dose decreased due to worsening of apathy |
| Tetrabenazine | | 8 | 8 | 3 | 2 | NA | Dose decreased due to suicidal thoughts, depression, parkinsonism |
| Antipsychotics | | 30 | 36 | 6 | 15 | 8 | Dose decreased due to apathy |
|  | Olanzapine | 12 | 16 | 2 | 6 | NA |  |
|  | Amisulpirid | 7 | 9 | 0 | 3 | NA |  |
|  | Risperidone | 8 | 12 | 2 | 6 | NA |  |
|  | Quetiapine | 6 | 2 | 2 | 0 | NA |  |
| Benzodiazepines | | 20 | 23 | 1 | 1 | 2 |  |
| Antidepressants | | 24 | 26 | 7 | 0 | 1 |  |
| Amantadine | | 17 | 10 | 0 | 4 | NA | Dose decreased due to agitation |
| Levodopa | | 2 | 1 | 0 | 1 | NA | Dose decreased due to worsening of cognition |

Number refers to admissions. Abbreviation: NA, not applicable. Note: Some patients had more than one antipsychotic at admission.

Supplementary Table 3: Further evaluation of hospitalized HD patients

| ECGs | 49 (36.3%) |
| --- | --- |
| Imaging  X-ray  CT  Sonography  MRI  Other (e.g., colonoscopy) | 33 (24.4%)  15 (11.1%)  9 (6.7%)  8 (5.9%)  6 (4.4%) |
| Consultation  None at all  Neuropsychologist (cognitive assessment)  Department of Hearing, Speech and Voice Disorders  Neurology  Psychiatry  Internal Medicine  Dentistry, oral surgery  Traumatology, Orthopaedics  ENT specialist  Ophtalmology  Dermatology  Psychology  Gynaecology  Neurosurgery  Surgery, aesthetic plastic surgery | 84 (62.2%)  15 (11.1%)  13 (9.6%)  8 (5.9%)  7 (5.2%)  6 (4.4%)  6 (4.4%)  5 (3.7%)  5 (3.7%)  5 (3.7%)  3 (2.2%)  3 (2.2%)  2 (1.5%)  2 (1.5%)  2 (1.5%) |
| Other assessments and clinical tests  (e.g., EEG, polysomnography) | 9 (6.7%) |

For categorical data the number and percent (%) are given. Abbreviations: ECG, electrocardiogram; X-ray, Energetic High-Frequency Electromagnetic Radiation; CT, computed tomography; MRI, magnetic resonance imaging; ENT, ear, nose and throat; EEG, electroencephalogram. Some patients had more than one consultation of another specialty.

**Reference**

1. Orth M, European Huntington's Disease N, Handley OJ, et al. Observing Huntington's disease: the European Huntington's Disease Network's REGISTRY. J Neurol Neurosurg Psychiatry 2011;82(12):1409-1412.
